# Anthropometric and motor-fitness signatures of defensive efficiency in professional football defenders: a principal component and cluster analysis

**DOI:** 10.1186/s13102-026-01711-y

**Published:** 2026-05-06

**Authors:** Jeneviv Nene John, Sam Chidi Ibeneme, Gerhard Fortwengel, Emeka Mong, Davidson Okwudili John, Ezinne Olive Nwosu, Blessing Chidimma Okpagu, Nnenna Christiana Chinagozi-Amanze, Paul Onyeudo

**Affiliations:** 1 Department of Medical Rehabilitation, Faculty of Health Sciences & Technology, University of Nigeria, Enugu Campus, Enugu State, Nigeria; 2 International Institute of Sports Research Development & Rehabilitation, David Umahi Federal University of Health Sciences, Uburu, Ebonyi State Nigeria; 3 Department of Physiotherapy, Faculty of Health Sciences, David Umahi Federal University of Health Sciences, Uburu, Ebonyi State Nigeria; 4 Department of Physiotherapy, Faculty of Health Sciences, School of Therapeutic Studies, University of the Witwatersrand, 7 York Road, Parktown, Johannesburg, Gauteng 2193 South Africa; 5 Faculty III, Hochschule Hannover University of Applied Sciences & Arts, Hannover, Germany; 6 Department of Human Kinetics & Health Education, Faculty of Education, Ebonyi State University, Abakaliki, Ebonyi State Nigeria; 7 Department of Physiotherapy, University of Nigeria Teaching Hospital, Ituku- Ozalla, Enugu, Enugu State Nigeria; 8 Department of Medical Laboratory Science, Faculty of Health Sciences & Technology, University of Nigeria, Enugu Campus, Enugu State, Nigeria; 9 Praxis für ganzheitliche, Physiotherapie, Eichendorffstraße 5, Neckartailfingen, Baden-Württemberg, 72666 Germany; 10 Department of Sports Medicine, Federal Ministry of Sports Development, Federal Capital Territory, Abuja, Nigeria

**Keywords:** Team-level defensive efficiency, Anthropometry, Motor fitness, Football defenders, Nigeria Premier Football League, Ecological performance analysis

## Abstract

**Background:**

Team-level defensive efficiency is a central determinant of success in professional football, yet defenders’ physical and motor profiles are rarely examined in relation to ecological defensive outcomes, particularly in under-researched professional leagues. This study investigated anthropometric and motor-fitness signatures of defensive efficiency among professional football defenders in the Nigeria Premier Football League (NPFL) using multivariate profiling methods.

**Methods:**

An observational ecological study was conducted among 36 professional defenders from Enugu Rangers International FC across three competitive seasons (2021/22–2023/24). Standardised anthropometric and motor-fitness assessments (agility, vertical-jump power, reaction time, balance, and coordination) were obtained during pre-season testing. Team-level defensive efficiency was derived from predefined ecological defensive indicators obtained from official league statistics, including goals conceded per match, defensive success index, points per goal conceded, and goal-prevention rate. Analyses included Spearman correlations, Kruskal–Wallis tests with eta-squared effect sizes (η²_H), principal component analysis (PCA), and k-means clustering; no individual-level regression was undertaken.

**Results:**

GA/PLD was relatively stable between full seasons (0.79 in 2021/22; 0.87 in 2023/24), whereas DSI and PPGC improved (0.68→0.74; 1.87→2.06), with GPR and GD/PLD also increasing (0.27→0.38; 0.29→0.53), indicating that comparable goals conceded yielded more favourable results. Defenders showed substantial muscularity (mean BMI 25.7 kg/m²; muscle mass 41.2 kg) and good motor fitness (agility 11.0 s). Anthropometric variables were strongly coupled, and BMI and jump classifications showed large effects on muscle mass, body fat, and jump power. PCA yielded three components explaining 72.5% of variance (body size/composition; agility–balance–coordination; power vs. adiposity), and k-means clustering identified three defender archetypes that differed most clearly in agility, separating heavier, slower stoppers from leaner, faster coverage profiles and balanced hybrids.

**Conclusion:**

This ecological profiling study shows that, within a single NPFL club, professional defenders cluster into distinct anthropometric and motor-fitness archetypes—ranging from heavier high-mass stoppers to leaner, more agile mobile coverage defenders—while team-level indicators suggest increasingly efficient conversion of broadly stable goals conceded into better results and goal difference. These patterns do not establish individual causal effects but illustrate how multivariate profiling can support role allocation, interpretation of body-size and power metrics, and context-sensitive conditioning in data-limited professional environments. Inferences remain exploratory and are constrained by single-club sampling, lack of player-level event data, and unquantified cluster stability.

**Supplementary Information:**

The online version contains supplementary material available at 10.1186/s13102-026-01711-y.

## Background of the study

Team-level defensive efficiency is a critical determinant of success in elite football, shaping match outcomes, tactical stability, and long-term league standing [1–3]. Teams that consistently limit goals conceded, maintain clean sheets, and protect narrow leads gain a structural advantage over the course of a season [2, 4]. Modern match analysis has tended to emphasise attacking metrics such as goals, expected goals and shot quality, yet defensive organisation, including how effectively a team prevents shots, protects dangerous spaces, and withstands pressure, is equally central to competitive success [1, 3, 5]. In many professional environments, especially outside the major European leagues, the absence of comprehensive tracking systems and detailed event coding results in defensive quality being inferred indirectly from team outcomes such as goals conceded per match, points gained, and goal difference rather than from fine-grained defensive actions [1, 5–8]. This ecological use of team-level indicators is now common in performance analysis when reliable event-level defensive data are unavailable or incomplete [2, 6].

A growing body of work has characterised the anthropometric profiles of professional footballers and documented systematic differences across playing positions [9–14]. Defenders generally present greater body mass and muscle mass than midfielders and wide attackers, reflecting the physical demands of duels, aerial contests and contact play near the defensive third [11–14]. Studies of elite and sub-elite players have shown that sprint speed, agility, strength, power and body composition are key correlates of football performance, and that positional roles are associated with distinct fitness requirements [9, 12, 14–20]. Longitudinal and developmental research further indicates that growth, maturation and body-composition trajectories influence role specialisation and progression into professional squads [15, 20]. Recent performance-analysis and Key performance indicator (KPI) frameworks now routinely employ indicators such as goals conceded, clean sheets and defensive pressures as markers of team defensive resilience and organisation [1, 2, 5]. Within this context, contemporary performance-analysis frameworks employ outcome-based defensive key performance indicators as proxies for team defensive resilience and organisation. Such indicators are widely used to characterise team-level defensive efficiency, particularly when detailed event-level data are unavailable [16, 21–23]. Principal component and cluster analyses have been used to derive practically interpretable archetypes that integrate several physical and neuromotor dimensions into coherent player profiles [21–23].

Despite this progress, several important gaps remain. First, the majority of position-specific profiling studies have been conducted in European or highly resourced environments, with limited representation of African professional leagues such as the Nigeria Premier Football League (NPFL) [9, 12–14, 16, 20, 24]. Second, most existing work on performance prediction has focused on attacking output or global match success, while defensive efficiency particularly for defenders as a specialist sub-group has received comparatively less attention [1–3, 5, 14]. Third, where defensive actions are analysed, they often rely on event-level data (tackles, interceptions, blocks, pressures) that are not consistently available in leagues without comprehensive tracking infrastructure [1, 3, 6–8]. This limits the transfer of advanced defensive analytics into applied practice in such contexts and makes it difficult for clubs in data-constrained environments to apply the same modelling frameworks used in major European competitions [7, 8, 17, 25]. Finally, there is a lack of ecological modelling approaches that explicitly link defenders’ anthropometric and motor-fitness profiles to ecological defensive indicators (e.g. goals conceded per match, defensive success indices) across competitive seasons, and that use multivariate methods (e.g. principal component analysis, clustering) to derive practically interpretable defender archetypes [21–23].

Accordingly, this study adopts an ecological profiling approach focused specifically on professional defenders in a Nigerian elite club context. Building on contemporary views that player performance should be interpreted in relation to their interaction with the competitive environment and match demands [1, 3, 7], this study links defenders’ anthropometric and motor-fitness characteristics to robust team-level defensive indicators derived from official NPFL match statistics. Rather than attempting unsupported individual-level causal modelling, we sought to (i) describe the anthropometric and motor-fitness profiles of professional defenders within a single NPFL club, (ii) quantify team-level defensive efficiency across multiple seasons using ecologically meaningful indicators, and (iii) identify latent physical–performance dimensions and defender archetypes using principal component and cluster analyses [21–23]. These objectives are descriptive and exploratory; the study does not attempt to model causal effects of individual defender profiles on defensive outcomes.

This multivariate, ecology-aware design represents a novel application of profiling methods to defensive efficiency in an African professional setting, where rich tracking data are limited but high-quality testing and official match statistics are available [7, 8, 17, 25]. To achieve these aims, we conducted an observational ecological study of professional defenders from Enugu Rangers International FC across three competitive seasons. Standardised anthropometric and motor-fitness assessments (including agility, reaction time, balance, coordination and jump-derived power) were combined with team-level defensive indicators computed from verified league data (goals against, matches played, results and points). We then used a sequence of non-parametric correlation and group-comparison tests, principal component analysis to identify core physical–performance dimensions, and k-means clustering to derive defender archetypes [21–23]. By integrating these elements, the study provides a practical, data-driven framework for understanding how defenders’ physical and neuromotor profiles align with team-level defensive efficiency as characterised by ecological defensive indicators in a resource-constrained professional environment and offers a template for similar analyses in other leagues lacking comprehensive tracking systems [7, 8, 25, 26]. While similar positional profiling frameworks have been described in European professional contexts, their application within an elite Nigerian club using ecological defensive indicators represents a contextual extension rather than a fundamentally new conceptual model.

## Methods

### Study design and setting

This observational ecological study was conducted among professional football defenders from Enugu Rangers International Football Club, a Nigeria Premier Football League (NPFL) team competing at the highest national level, across three competitive seasons. Anthropometric and motor-fitness assessments were conducted during structured pre-season testing sessions prior to the commencement of each competitive season. Testing procedures were conducted at the start of the 2021/22, 2022/23, and 2023/24 NPFL seasons using the same standardised protocols and with all assessments performed by the same assessor. Because detailed individual defensive event data (e.g. tackles, interceptions, blocks, pressures) are not systematically recorded in the NPFL, season-level defensive outcomes (e.g. goals conceded per match, defensive success indices and points per goal conceded) were used as ecological proxies for defensive performance rather than individual defensive events.

### Participants

The target population comprised thirty-six professional defenders registered with Enugu Rangers International FC. For this study, “defender” was defined as players whose primary position was central defender, full-back or wing-back in the club’s official squad lists and tactical reports for at least one full NPFL season during the study period.

Inclusion criteria were:

1. Registered as a defender for Enugu Rangers in at least one of the seasons of interest (2021/22, 2022/23 or 2023/24).
2. Participation in ≥ 50% of league fixtures in at least one season (e.g. ≥19 matches in a 38-match season).
3. Completion of the full anthropometric and motor-fitness testing battery during pre-season assessments.

Exclusion criteria were:

1. Long-term injury or medical condition precluding participation in physical testing.
2. Incomplete or missing data for key anthropometric or motor-fitness variables.
3. Sustained reassignment to a non-defensive role (e.g. conversion to full-time midfielder) during the observation period.

Eligibility required participation in ≥ 50% of league fixtures in at least one season (e.g. ≥19 matches in a 38-match season), ensuring that included defenders were regular contributors rather than peripheral squad members. Detailed match-exposure metrics (e.g. minutes played, starter vs. substitute status) were not consistently available across all seasons and were therefore not analysed further, in line with the ecological design of the study. All players were members of the senior squad or its affiliated development squad and trained within the same high-performance environment, strength-and-conditioning programme and tactical framework.

### Anthropometric assessment

Anthropometric assessments were conducted by experienced physiotherapists and sport-science staff, using standardised protocols across the three seasons [27]. Body mass was measured to the nearest 0.1 kg using a calibrated digital scale with players wearing light clothing and no footwear. Standing height was measured to the nearest 0.1 cm using a wall-mounted stadiometer. Body mass index (BMI) was calculated as weight (kg) divided by height squared (m²).

Circumferential measures included waist and hip circumference, recorded to the nearest 0.1 cm with a non-elastic tape at standard anatomical landmarks [27]. Waist-hip ratio (WHR) was calculated as waist circumference divided by hip circumference.

Body composition (percentage body fat and muscle mass) was estimated using a multi-frequency bioelectrical impedance analyser according to the manufacturer’s recommendations. Measurements were taken in the morning under controlled conditions (no heavy meal or vigorous exercise in the preceding hours) [28]. From these data, defenders were categorised into BMI and, where relevant, body-fat classes for descriptive and group-comparison purposes [9, 14].

### Motor-fitness assessments

Motor-fitness testing was conducted by the same assessor on the club’s training pitch and indoor facility during pre-season, under stable environmental conditions and after a standardised warm-up (light running, dynamic stretching and submaximal practice trials). All players were familiar with the tests through routine performance monitoring. In the present study, motor fitness is conceptualised as a multidimensional construct encompassing agility, balance, coordination, and reaction time, attributes that underpin the efficient execution of defensive tasks in professional football.

#### Agility (T-Test)

Change-of-direction ability was assessed using a standard T-Test agility protocol [29]. Players sprinted forward, shuffled laterally, and back-pedalled through a cone-defined “T” pattern. Time to completion was recorded to the nearest 0.01s using handheld electronic stopwatches operated by trained assessors. Lower times indicated better agility.

#### Vertical-jump power

Lower-limb explosive power was estimated using a countermovement vertical jump [30]. Jump height was obtained using a jump mat or contact system and converted to mechanical power (watts) via a validated regression equation incorporating standing height and jump height [31]. Players performed two to three maximal trials with adequate rest; the best trial was retained.

#### Reaction time

Simple reaction time was assessed using a standard vertical-drop device (e.g. ruler-drop or electronic drop apparatus) [32]. Players were instructed to respond as quickly as possible to an unpredictable release; reaction time was calculated in milliseconds from the distance fallen or device output. The mean of repeated trials was used as the performance measure.

#### Dynamic balance (Y-Balance Test)

Dynamic postural control was assessed using the Y-Balance Test [33, 34]. From a single-leg stance on the test platform, players reached in the anterior, posteromedial, and posterolateral directions along marked lines. Each direction was performed three times, with the maximal reach distance recorded. Composite reach scores were calculated as the sum of the three reach directions, normalised to limb length, and expressed as a percentage of limb length [33, 34]. Higher composite scores indicated better dynamic balance performance.

#### Coordination (wall-volley test)

Football coordination was assessed using a timed wall-volley task, a commonly used field-based coordination and ball-control assessment in football [35, 36]. Defenders stood at a fixed distance from a vertical wall and repeatedly volleyed a standard ball towards a marked target square for a fixed period (30s). Successful on-target returns were counted; the highest of two trials was retained. All tests were supervised by the same staff across seasons, with standardised instructions and rest intervals to minimise fatigue effects.

### Ecological defensive indicators

Team-level defensive efficiency was characterised using predefined ecological defensive indicators derived from official NPFL seasonal statistics corresponding to defender involvement. For each season (2021/22, 2022/23 and 2023/24), match data were extracted from verified public football statistics databases and cross-checked across sources [37, 38]. The following season-level variables were recorded: matches played (PLD), wins (W), draws (D), goals for (GF), goals against (GA), and points (PTS).

From these, five ecological defensive indicators were computed. These indicators are widely used in football performance analysis to characterise defensive efficiency, match control and competitive success [2, 4, 6].

#### Goals Conceded per Match (GA/PLD)

$${\mathrm{GA/PLD}}=\frac{\mathrm{Goals}\:\mathrm{Against}}{\mathrm{Matches}\:\mathrm{Played}}$$

Widely used as a core indicator of defensive efficiency in football performance analysis, this metric reflects higher defensive efficiency when values are lower [2, 4].

#### Defensive Success Index (DSI)

$${\mathrm{DSI}}=\frac{\mathrm{W}\:+\:\mathrm{D}}{\mathrm{P}\mathrm{L}\mathrm{D}}$$

Reflects the proportion of matches not lost and provides an outcome-based proxy for defensive stability and match control [3, 39].

#### Points per Goal Conceded (PPGC)

$${\mathrm{PPGC}}=\frac{\mathrm{PTS}}{\mathrm{GA}}$$

Quantifies the relationship between goals conceded and league points, capturing the contribution of defensive stability to competitive success [3, 40].

#### Goal-Prevention Rate (GPR)

   

$${\mathrm{GPR}}=1-\frac{\mathrm{GA}}{GF}\;{\mathrm{when}}\;{\mathrm{GF}}>0$$

Reflects team-level defensive efficiency relative to attacking output by contextualising goals conceded against goals scored, consistent with approaches examining attack–defence balance in match outcomes [6, 26, 41].

#### Goal Difference per Match (GD/PLD)

$$\:\frac{\mathrm{G}\mathrm{D}}{\mathrm{P}\mathrm{L}\mathrm{D}}\:=\:\frac{\mathrm{G}\mathrm{F}-\mathrm{G}\mathrm{A}}{\mathrm{P}\mathrm{L}\mathrm{D}}$$

Provides contextual insight into net scoring margin attributable in part to team-level defensive efficiency [2, 4]. This metric reflects overall performance sustainability and indirectly captures defensive solidity when GA is low.

The definitions, computational formulas, and analytical interpretation of all ecological defensive indicators used in this study are summarised in Table 1.

**Table 1:** Summary of Ecological Defensive Indicators, Computational Formulas, and Intended Analytical Use

Indicators	Formula	Intended Use / Interpretation
Goals Conceded per Match (GA/PLD)	$\:\frac{\mathrm{G}\mathrm{A}}{\mathrm{P}\mathrm{L}\mathrm{D}}$	Primary defensive-efficiency metric. Lower values indicate stronger defensive organization and goal-prevention capacity. Used as the main dependent variable in modelling.
Defensive Success Index (DSI)	$\:\frac{\mathrm{W}\:+\:\mathrm{D}}{\mathrm{P}\mathrm{L}\mathrm{D}}$	Measures the proportion of matches not lost. Reflects defensive stability, tactical cohesion, and ability to maintain positive match outcomes.
Points per Goal Conceded (PPGC)	$\:\frac{\mathrm{P}\mathrm{T}\mathrm{S}}{\mathrm{G}\mathrm{A}}$	Indicates how effectively defensive solidity contributes to league points. Higher values imply efficient conversion of team-level defensive efficiency into competitive advantage
Goal-Prevention Rate (GPR)	1 - $\:\frac{\mathrm{G}\mathrm{A}}{\mathrm{G}\mathrm{F}}$ when GF > 0	Contextual metric showing defensive strength relative to attacking output. Helps evaluate balance between defensive resilience and offensive productivity.
Goal Difference per Match (GD/PLD)	$\:\frac{\mathrm{G}\mathrm{F}-\mathrm{G}\mathrm{A}}{\mathrm{P}\mathrm{L}\mathrm{D}}$	Provides net scoring margin per match. Not purely defensive but offers contextual insight into match performance sustainability. Used for sensitivity and descriptive analysis.

The 2022/23 season, for which only 18 league matches were recorded in the available data, was treated descriptively and excluded from longitudinal trend inferences because of probable incompleteness. Defensive indicators from 2021/22 to 2023/24 were therefore emphasised when interpreting multi-season patterns. These indicators served as ecological, season-level descriptors rather than individual-level outcomes and were used to contextualise defender profiles rather than to perform player-level regression modelling.

### Data management

All anthropometric and motor-fitness data were entered into a secure spreadsheet and checked for completeness and outliers. Consistency checks were performed by cross-referencing original field forms and electronic entries. Season-level defensive statistics were imported from verified online databases and cross-validated against independent sources. Identifiers were removed and replaced by study codes prior to analysis.

### Statistical analysis

All statistical analyses were performed using IBM SPSS Statistics version 29.0 (IBM Corp., Armonk, NY, USA) and R version 4.3.2 (R Foundation for Statistical Computing, Vienna, Austria). PCA and k-means clustering were implemented in R using the packages FactoMineR and factoextra, with additional visualisation via ggplot2. The significance threshold was set at *p* < 0.05, but *p*-values were interpreted alongside effect sizes and the exploratory nature of the study. Given the ecological level of the defensive indicators and sample-size constraints, no multivariable regression models were fitted linking individual players directly to team-level defensive outcomes.

#### Normality and preliminary checks

Distributional assumptions were examined using Shapiro–Wilk tests and inspection of Q–Q plots. As most variables deviated significantly from normality, non-parametric procedures were adopted for correlation and group comparisons. Full Shapiro–Wilk results are presented in Supplementary Table S1. All analyses were exploratory and ecological in nature, designed to profile defenders’ characteristics rather than to predict individual defensive events.

#### Correlation analysis

Bivariate associations among anthropometric and motor-fitness variables were explored using Spearman rank-order correlation coefficients. Correlation matrices were examined to identify strong linear and monotonic relationships, and to inform subsequent multivariate analysis and interpretation.

#### Group comparisons

Group differences were evaluated using Kruskal-Wallis tests with Bonferroni-adjusted post-hoc pairwise comparisons across predefined tertile-based classifications for BMI, agility, and vertical jump power, as well as across defender archetype clusters identified in the multivariate analysis. These tests were used to examine differences in anthropometric and motor-fitness variables across the predefined classifications. For each Kruskal-Wallis test, effect size was quantified as η²_h (eta-squared based on H), providing an estimate of the proportion of variance in ranks attributable to the grouping factor. Where global tests were significant, post-hoc pairwise comparisons were performed using Mann-Whitney U (Wilcoxon rank-sum) tests with Bonferroni-adjusted *p*-values. Effect size r for pairwise contrasts was calculated as r = Z/√N, where Z is the standardised test statistic and N is the total sample size for the pairwise comparison.

#### Principal component analysis (PCA)

To reduce dimensionality and identify latent physical–performance dimensions, PCA was applied to a set of continuous anthropometric and motor-fitness variables. All variables were standardised (z-scores) prior to analysis to place them on a common scale. Components with eigenvalues > 1, inspection of scree plots and cumulative variance explained were used to determine the number of retained components. Component loadings were examined to interpret each principal component (e.g. body size/composition, agility–balance–coordination, power vs. adiposity). Principal component analysis (PCA) was applied for exploratory profiling to identify latent physical–performance dimensions; components were interpreted descriptively and not used for predictive modelling.

#### Cluster analysis

K-means clustering was applied to the retained principal component scores to derive defender archetypes. The number of clusters (k) was selected based on interpretability, inspection of within-cluster sum-of-squares plots (elbow method), and clarity of centroid separation in principal-component space. Given the modest sample size, no formal resampling-based cluster stability analysis was conducted; the three-cluster solution should therefore be regarded as provisional and sample-specific. Cluster centroids and distributions of key variables across clusters were examined to characterise archetypes (e.g. high-mass stoppers, mobile coverage defenders, balanced hybrids). Kruskal–Wallis and post-hoc tests were subsequently used to assess differences between clusters in key anthropometric and motor-fitness outcomes.

There were no missing values in the variables included in the multivariate analyses. Given the ecological level of the defensive indicators and sample-size constraints, no multivariable regression models were fitted linking individual players directly to team-level defensive outcomes.

### Ethical considerations

The study protocol was reviewed and approved by the University of Nigeria Health Research Ethics Committee (NHREC/05/01/2008B-FWA0000245 8-1RB00002323), with specific renewal for the period covering the 2021–2023 data-collection cycles. All procedures conformed to the principles of the Declaration of Helsinki. Players received verbal and written information about the study aims and procedures and provided written informed consent prior to participation. Club management granted permission for use of anonymised performance and match-statistics data for research purposes.

## Results

### Descriptive statistics

Table 2 presents the descriptive statistics for the anthropometric and motor-fitness profiles of the 36 professional defenders, with a mean age of 23.06 years (SD = 2.13; range = 20–27 years), indicating a relatively young but mature professional cohort.

- Objective 1: anthropometric and motor-fitness profiles of professional defenders within a single NPFL club

**Table 2:** Descriptive Statistics for Anthropometric and Motor-Fitness profiles

Variable	M	SD	Min	Max
Age (years)	23.06	2.13	20.00	27.00
Height (m)	1.72	0.07	1.61	1.84
Weight (kg)	76.53	7.27	65.00	88.00
BMI (kg/m²)	25.68	1.05	24.02	27.77
% Body Fat	16.32	9.17	8.30	31.70
Muscle Mass (kg)	41.15	4.52	33.80	46.20
Coordination (seconds)	26.57	1.74	24.00	30.00
Agility (s)	11.04	0.95	9.50	13.50
Y-Balance (cm)	96.87	12.18	77.70	115.90
Vertical Jump Power (W)	3710.71	284.79	3325.00	4286.00
Reaction Time (ms)	94.29	17.89	70.00	120.00

Players had an average height of 1.72 m, body mass of 76.53 kg, a mean BMI of 25.68 kg/m² and mean muscle mass of 41.15 kg, indicating substantial muscular development. Motor fitness attributes were characterised by a mean agility time of 11.04 s and vertical jump power of 3710.71 watts. Reaction times were fast (M = 94.29 ms), and balance performance was high (M = 96.87 cm), reflecting good postural control and dynamic stability.

- Objective 2: team-level defensive efficiency across multiple seasons using ecological defensive indicators

### Team-level defensive performance

Ecological defensive indicators for Enugu Rangers across the three seasons are summarised in Tables 3 and 4. These metrics are team-level, season-aggregated indicators and do not represent individual defenders’ performance. The 2021/22 season recorded the lowest goals conceded per match (GA/PLD = 0.789), indicating the strongest raw defensive containment. However, overall team-level defensive efficiency improved over time, with the highest Defensive Success Index (DSI = 0.737) and Points per Goal Conceded (PPGC = 2.061) observed in the 2023/24 season. The Goal-Prevention Rate (GPR) also peaked in 2023/24 (0.377), reflecting an improved balance between defensive solidity and attacking output.

**Table 3:** Ecological Defensive Indicators Across Three NPFL Seasons for Enugu Rangers

Season	PLD	W	D	GF	GA	PTS	GA/PLD	DSI	PPGC	GPR	GD/PLD
2021/22	38	15	11	41	30	56	0.789	0.684	1.867	0.268	0.289
2022/23*	18	4	9	16	16	21	0.889	0.722	1.312	0.000	0.000
2023/24	38	20	8	53	33	68	0.868	0.737	2.061	0.377	0.526

*Key*: *PLD*  Matches Played, *W/D*  Wins / Draws, *GF/GA*  Goals For/Against, *PTS*  Points, *GA/PLD*  Goals Conceded per Match (lower is better), *DSI*  Defensive Success Index = (W + D) / PLD, *PPGC*  Points Per Goal Conceded = PTS / GA, *GPR*  Goal Prevention Rate = 1 - (GA / GF), *GD/PLD*  Goal Difference per Match

**Table 4:** Team-Level Defensive Indicators Across Seasons

Season	GA/PLD	DSI	PPGC	GPR	GD/PLD
2021/22	0.789	0.684	1.867	0.268	0.289
2022/23*	0.889	0.722	1.312	0.000	0.000
2023/24	0.868	0.737	2.061	0.377	0.526

*Key*: *GA/PLD*  Goals Conceded per Match (lower is better), *DSI*  Defensive Success Index = (W + D) / PLD, *PPGC*  Points Per Goal Conceded = PTS / GA, *GPR*  Goal Prevention Rate = 1 - (GA / GF), *GD/PLD*  Goal Difference per Match

The 2022/23 season, for which only 18 league matches were recorded, is treated as a truncated season: its indicators are presented descriptively but excluded from longitudinal trend interpretation because of probable incompleteness. Thus, multi-season inferences focus primarily on 2021/22 and 2023/24 (Tables 3 and 4).

- Objective three: Identify latent physical–performance dimensions and defender archetypes using principal component and cluster analyses

### Normality testing

Preliminary Shapiro-Wilk tests indicated that the majority of anthropometric and motor-fitness variables deviated from normal distribution assumptions (*p* < 0.05), with only agility and vertical jump power showing approximate alignment. Given these findings and the modest sample size, non-parametric statistical tests were adopted for subsequent group comparisons and association analyses. Full Shapiro–Wilk results are presented in Supplementary Table S1.

### Spearman correlation analysis

**Fig. 1:**
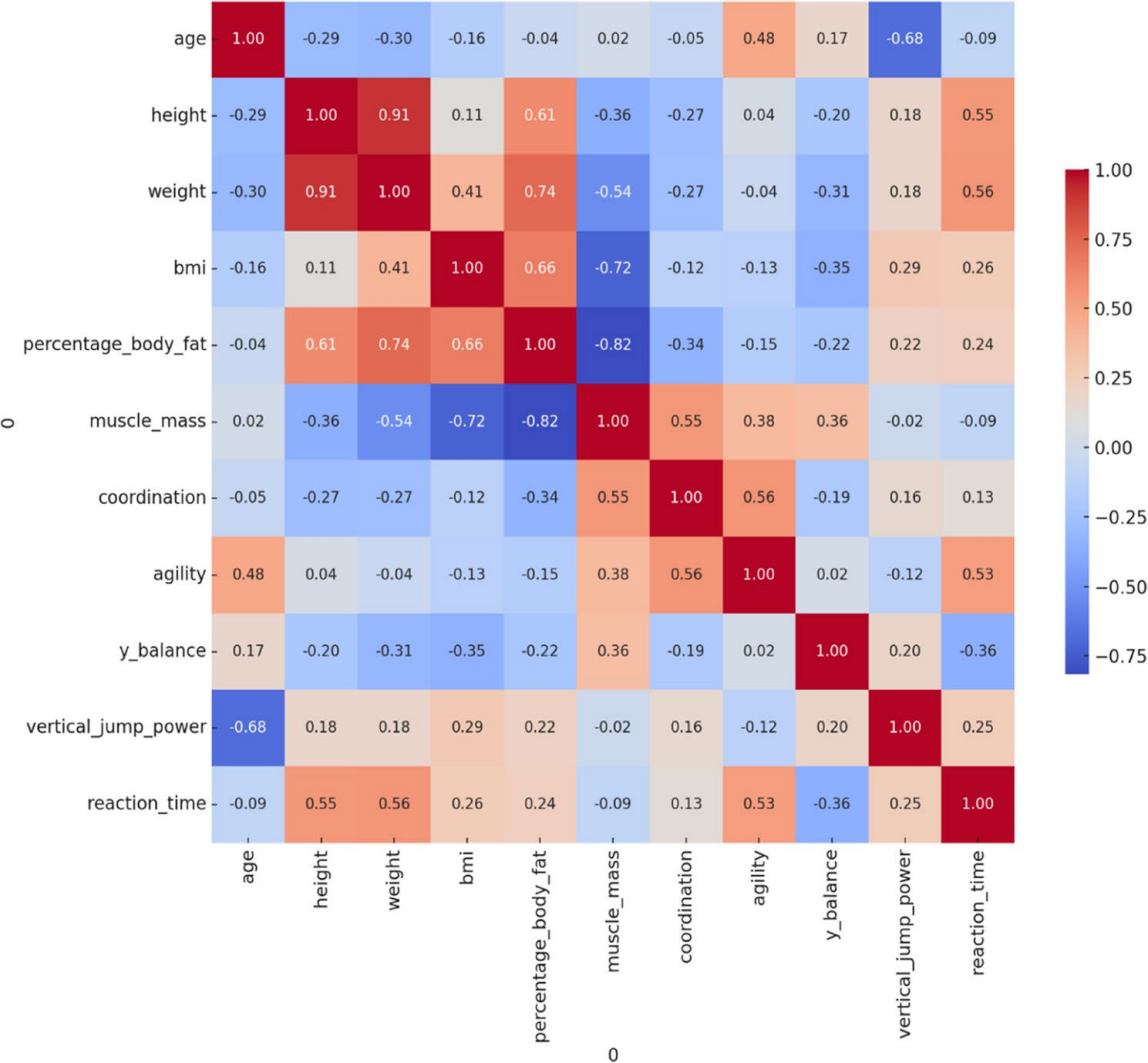
Spearman Correlation Heatmap

Spearman correlation coefficients (Fig. 1) revealed strong associations among anthropometric variables. Hip circumference correlated highly with both height (ρ = 0.93) and weight (ρ = 0.95). Waist circumference was strongly associated with percentage body fat (ρ = 0.77), and BMI showed a strong positive correlation with waist circumference (ρ = 0.74). Notable negative correlations were observed between waist-to-hip ratio (WHR) and hip circumference (ρ = − 0.92), indicating that higher hip circumference was associated with lower WHR. Agility showed moderate negative correlations with height (ρ = −0.56) and weight (ρ = −0.55), suggesting that taller and heavier defenders tended to record slower agility times. These correlations are descriptive and ecological in nature and were used to inform multivariate profiling rather than predictive modelling.

### Group comparisons: kruskal-wallis tests

Non-parametric Kruskal-Wallis tests (Table 5) were used to examine differences in anthropometric and motor fitness variables across categorical classifications. Key findings included:

- BMI classification significantly differentiated muscle mass (H = 11.88, *p* = 0.0006) and body fat percentage (H = 7.24, *p* = 0.0071). Corresponding effect sizes based on η²_H indicated medium-to-large and small-to-medium effects, respectively (muscle mass: η²_H ≈ 0.28; body fat: η²_H ≈ 0.15), consistent with greater muscle and fat levels in higher BMI categories.
- Vertical jump classification explained substantial variation in jump power (H = 17.54, *p* = 0.0002), with a large effect size (η²_H ≈ 0.44), supporting the validity of this categorisation for discriminating lower-limb power.
- Agility, Y-balance, and reaction time classifications did not show statistically significant differences in other anthropometric or motor-fitness variables (all *p* > 0.05), and the corresponding effect sizes were negligible (η²_H ≈ 0.00–0.02).
- Post-hoc pairwise comparisons (Table 6) confirmed significant agility differences between Clusters 1 and 2 (*p* = 0.021, *r* ≈ 0.45) and between Clusters 1 and 3 (*p* = 0.046, *r* ≈ 0.44), indicating medium-sized effects in both contrasts. In contrast, the agility difference between Clusters 2 and 3 was not statistically significant (*p* = 0.650, *r* ≈ 0.09), with a small effect size. These findings suggest that defenders in Cluster 2, characterised as mobile coverage defenders, were meaningfully faster than those in Cluster 1 (high-mass stoppers) and Cluster 3 (balanced hybrids), although distributions overlapped. Given the modest sample size, these *p*-values and effect sizes should be interpreted as exploratory indicators of magnitude rather than confirmatory evidence of population-level differences.

**Table 5:** Kruskal-Wallis Tests Comparing Group Classifications

Grouping variable (3 tertiles)	Outcome variable	H (df = 2)	*p*-value	Effect size (η²_H)
BMI classification	Muscle mass (kg)	11.878	0.0006	0.28
BMI classification	% body fat	7.237	0.0071	0.15
BMI classification	Vertical jump power (W)	0.000	1.000	0.00
Agility classification (T-test)	Reaction time (s)	2.612	0.270	0.02
Agility classification (T-test)	Y-balance composite score	2.432	0.296	0.01
Vertical-jump classification	Vertical jump power (W)	17.544	0.0002	0.44
Muscle-mass classification	Agility (s)	0.125	0.939	0.00
Waist–hip ratio classification	BMI (kg·m⁻²)	0.377	0.828	0.00

Kruskal–Wallis effect sizes were calculated as η²_H = (H – (k – 1)) / (N – 1), where H is the Kruskal-Wallis statistic, *k* = 3 groups, and *N* = 36 defenders. Values of η²_H ≈ 0.01, 0.06 and 0.14 can be interpreted as small, medium and large effects, respectively. In this sample, BMI classification showed medium-to-large effects on muscle mass (η²_H = 0.28) and percentage body fat (η²_H = 0.15), and vertical-jump classification showed a large effect on jump power (η²_H = 0.44), whereas all other tests had negligible effect sizes (η²_H ≈ 0.00–0.02)

**Table 6:** Post-hoc Pairwise Comparisons After Kruskal-Wallis

Comparison	Outcome Variable	*p*-Value (Bonferroni)	Significant?
Cluster 1 vs. 2	Agility	0.021	Yes
Cluster 1 vs. 3	Agility	0.046	Yes
Cluster 2 vs. 3	Agility	0.650	No

### Principal Component Analysis (PCA)

**Fig. 2:**
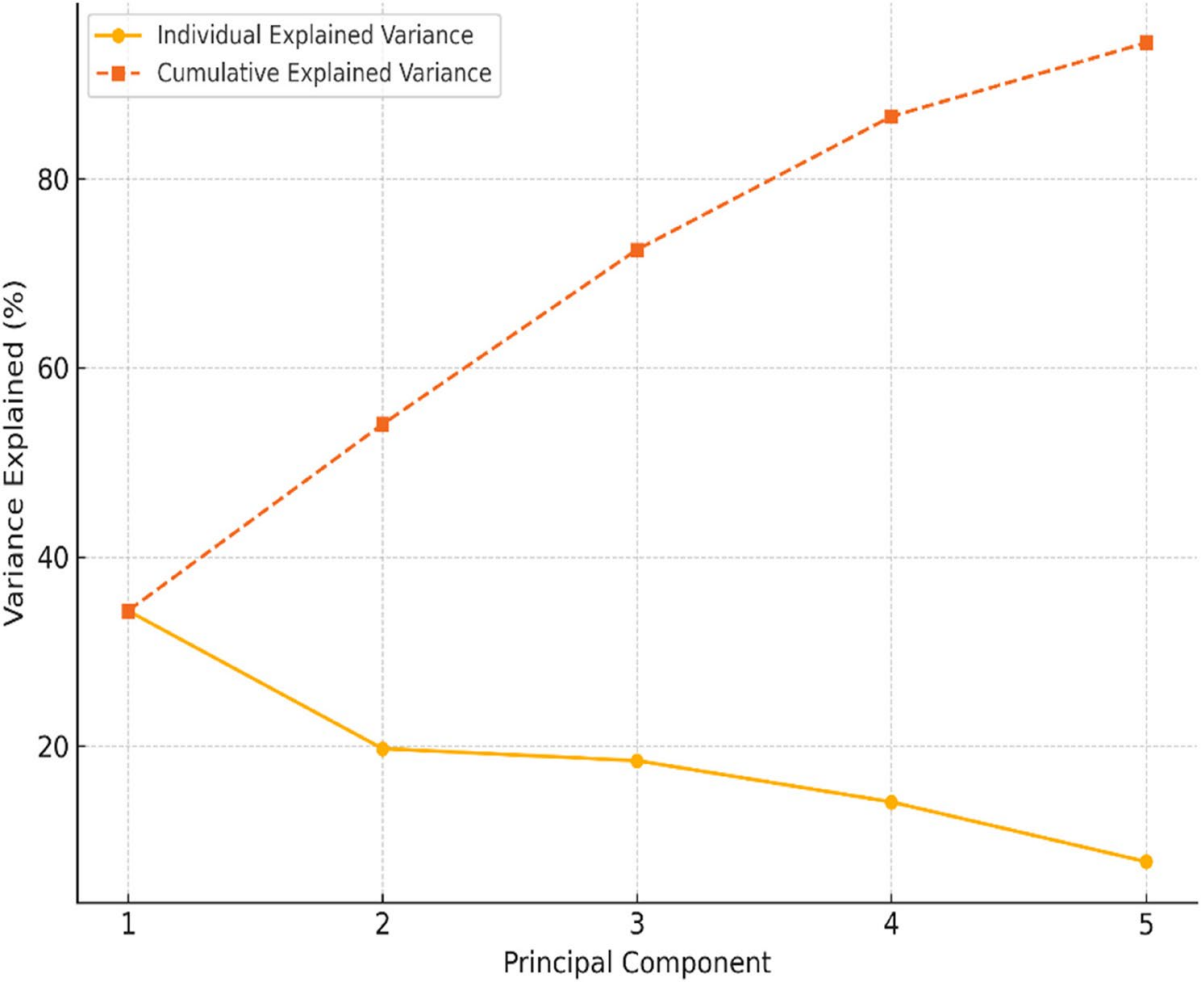
PCA scree plot showing variance explained by each principal component

To explore latent performance structures among defenders and reduce dimensionality, a PCA was conducted on 14 standardised continuous variables, including anthropometric and motor-fitness variables (Table 7). Figure 2 presents the PCA scree plot, which shows the proportion of variance explained by each principal component. The first three components had eigenvalues greater than 1 (Table 8) and were retained based on the Kaiser criterion and visual inspection of the elbow point in the plot (Fig. 2). These three components together explained 72.5% of the total variance (Table 9), supporting their inclusion in subsequent clustering and profiling analyses.

- PC1 (34.3% of variance) loaded strongly on height, weight, BMI, hip circumference and muscle mass, and was interpreted as a body size/composition dimension.
- PC2 (19.7%) reflected agility (negative loading), Y-balance and coordination, and was interpreted as an agility–balance–coordination component.
- PC3 (18.5%) showed high positive loadings for vertical jump power and negative loadings for body fat and reaction time, representing a power/explosiveness versus adiposity inverse dimension.

**Table 7:** PCA Loadings for Anthropometric and Motor-Fitness Variables

Variable	PC1	PC2	PC3
Height	0.86	-0.25	0.12
Weight	0.89	-0.18	0.20
BMI	0.82	-0.20	0.30
Hip Circumference	0.88	-0.19	0.18
Muscle Mass	0.79	-0.25	0.10
% Body Fat	0.35	-0.18	-0.72
Waist Circumference	0.67	-0.15	0.21
Waist–Hip Ratio	0.40	-0.08	-0.25
Agility	-0.22	-0.79	0.22
Y-Balance	-0.26	0.73	-0.10
Coordination	0.15	0.68	0.05
Vertical Jump Power	0.23	0.26	0.77
Reaction Time	0.19	-0.30	-0.67

**Table 8:** PCA Variance Explained Summary

Principal Component	Eigenvalue	Variance Explained (%)	Cumulative Variance (%)
PC1	3.884	34.300	34.300
PC2	2.234	19.730	54.030
PC3	2.090	18.460	72.490
PC4	1.596	14.090	86.580
PC5	0.881	7.780	94.360

**Table 9:** Scree plot of principal component analysis (PCA) showing variance explained by each component

Element	Figure 2	Text Description	Match?
PC1 Variance Explained	34.3%	*“PC1 (34.3%)”*	Yes
PC2 Variance Explained	19.7%	*“PC2 (19.7%)”*	Yes
PC3 Variance Explained	18.5%	*“PC3 (18.5%)”*	Yes
Cumulative Variance (PC1–3)	72.5%	*“first three principal components together explained 72.5%"*	Yes
Interpretations / Labels	Not shown on plot (visual only)	Text describes interpretation of PC1–PC3	Complementary

PCA was used for exploratory profiling rather than predictive modelling, and the components are interpreted descriptively within the ecological, single-club context. These components were used as input features for k-means clustering to identify broad defender archetypes. Detailed PCA loadings are presented in Table 7.

### K-means clustering and defender role profiling

**Fig. 3:**
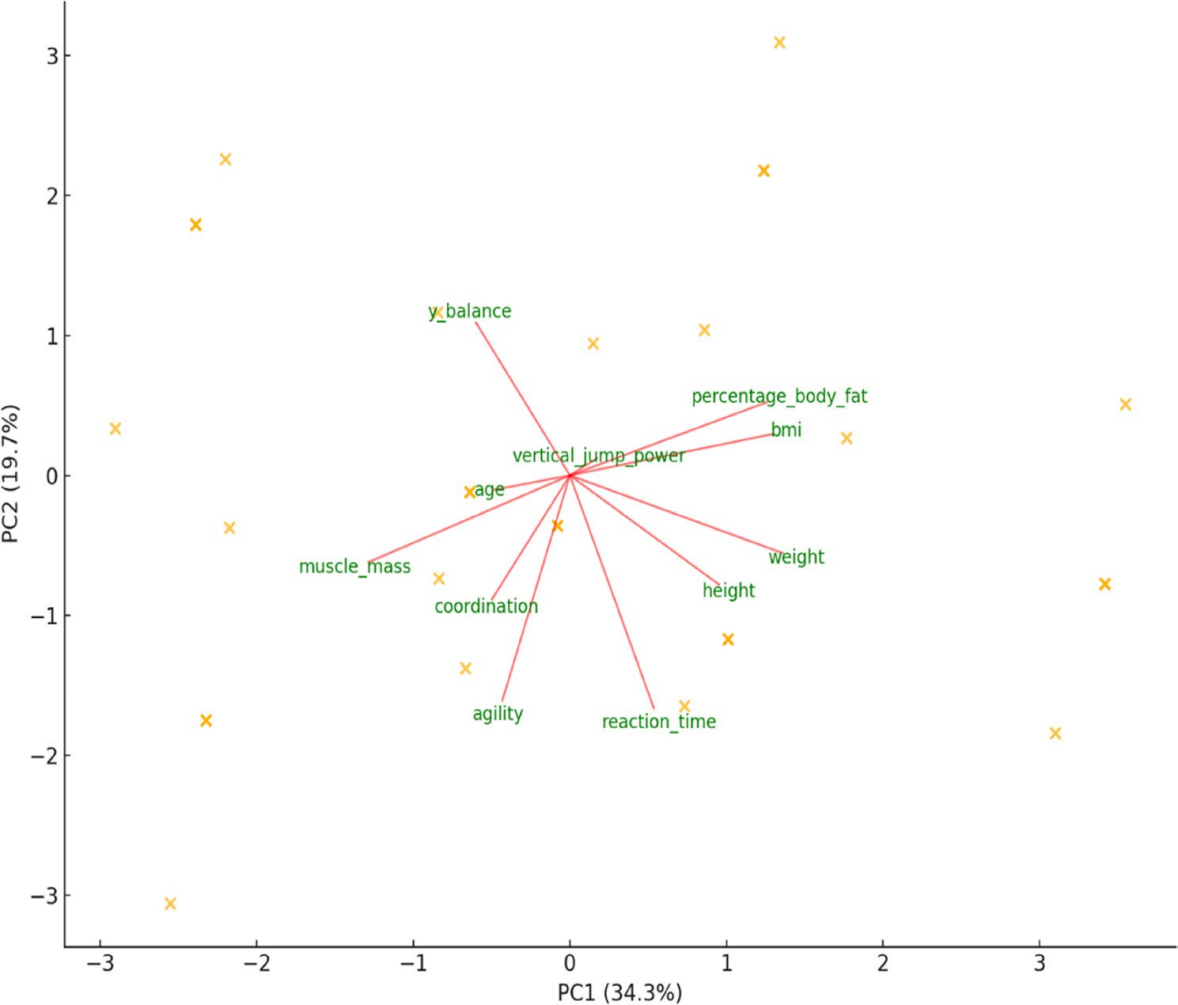
PCA cluster plot (PC1 vs. PC2) illustrating the spatial separation of defender archetypes

**Fig. 4:**
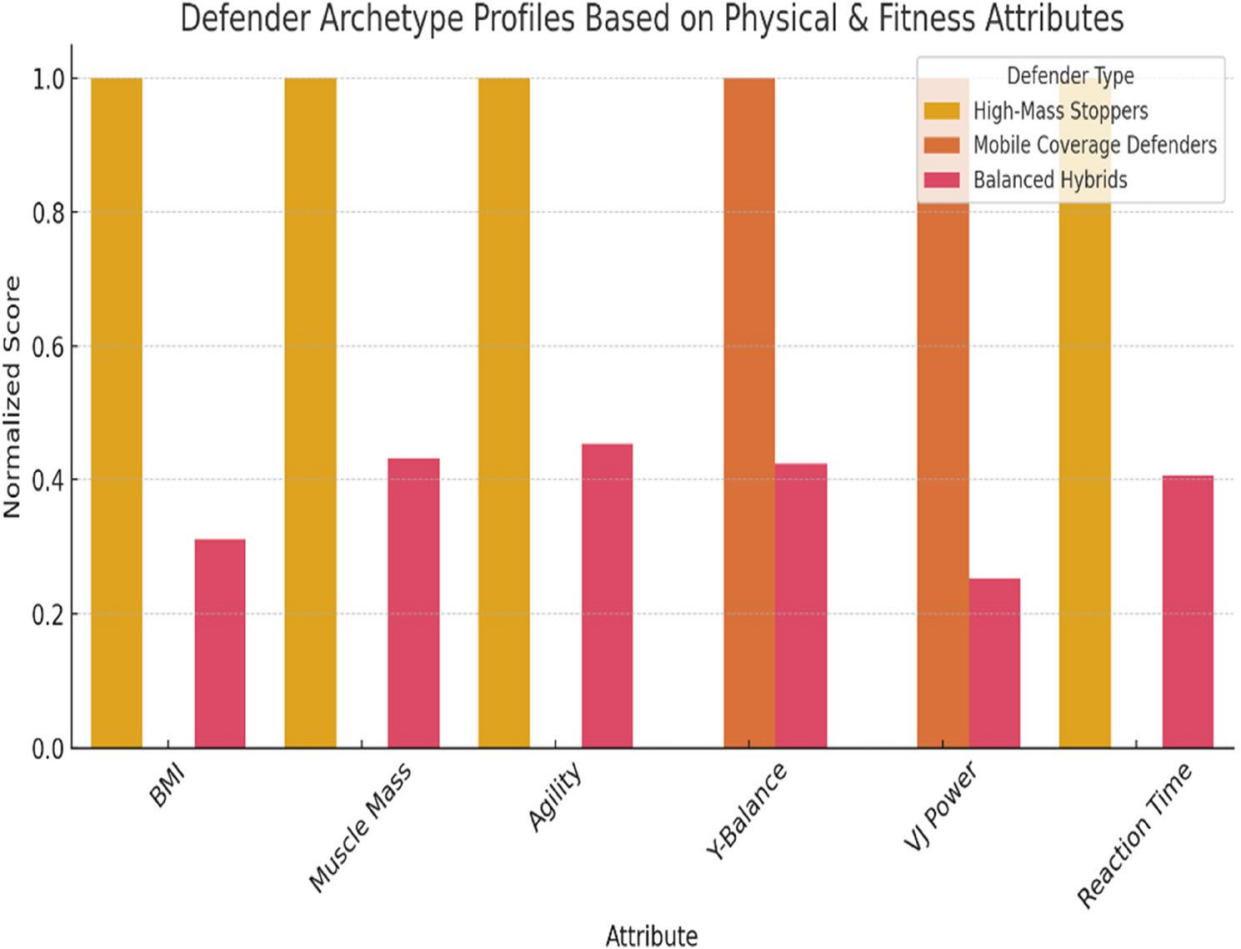
Archetype profile chart depicting average anthropometric and motor fitness characteristics for each cluster

Using scores from the first three principal components, k-means clustering identified three distinct defender archetypes (Figs. 3 and 4; Table 10):

- Cluster 1 (High-mass stoppers): Older defenders with higher BMI and muscle mass, and comparatively slower agility.
- Cluster 2 (Mobile coverage defenders): Younger, leaner defenders with faster agility, better balance, and relatively higher vertical jump power.
- Cluster 3 (Balanced hybrids): Players with intermediate values across most anthropometric and motor-fitness variables.

**Table 10:** Cluster-wise Summary of Key Anthropometric and Motor Fitnesss Attributes

Cluster	*n*	Age (M)	BMI (M)	Muscle Mass (M)	Agility (s)	Y-Balance (cm)	Vertical Jump Power (W)	Reaction Time (ms)
1 – High-Mass Stoppers	12	24.10	26.50	43.80	11.60	92.30	3625	98.20
2 – Mobile Coverage Defenders	14	22.50	24.90	38.70	10.50	101.50	3912	89.60
3 – Balanced Hybrids	9	23.30	25.40	40.90	11.00	96.20	3698	93.10

Kruskal–Wallis tests confirmed significant between-cluster differences in agility performance (*p* < 0.05), with a moderate between-cluster effect size for agility (η²_H ≈ 0.11), consistent with the descriptive agility differences shown in Table 10. Post-hoc tests showed that Cluster 2 was significantly faster than Clusters 1 and 3 (Table 6), supporting the interpretation of Cluster 2 as a more mobile, coverage-oriented profile. However, there was still substantial overlap in agility and other variables between clusters, consistent with continuous variation rather than rigid categorical splits. Cluster-wise distributions of key anthropometric and motor-fitness variables are summarised in Table 10.

Given the sample size and the exploratory nature of the k-means procedure, no formal resampling-based stability analysis (e.g. bootstrapping or cross-validation) was conducted. Consequently, the three-cluster structure should be interpreted as a plausible but provisional ecological classification rather than a definitive taxonomy of defender types, and its generalisability beyond this single NPFL club remains uncertain.

## Discussion

### Summary of key findings

This exploratory, ecological study profiled the anthropometric and motor-fitness characteristics of professional football defenders from a single Nigerian Premier Football League (NPFL) club and related these profiles descriptively to team-level defensive indicators across three competitive seasons. Defenders showed substantial variation in body size, body composition and motor fitness despite operating within the same professional environment. Principal component analysis reduced these multidimensional attributes to three interpretable components reflecting (i) body size and mass, (ii) agility–balance–coordination, and (iii) explosiveness contrasted with adiposity and slower reaction time. Subsequent k-means clustering identified three defender archetypes: higher mass “stoppers”, more mobile “coverage defenders”, and “balanced hybrids” that differed primarily in agility and vertical jump power. Agility and vertical jump power showed at least medium effects between clusters, whereas other contrasts showed negligible effects, underscoring the exploratory nature of the observed patterns. At the ecological level, defensive indicators (e.g. goals conceded per match, defensive success index, goal-prevention rate, points per goal conceded) suggested relatively stable and efficient defensive organisation over time within the club. Importantly, the study does not seek to attribute these team-level outcomes to specific player types, but rather to describe how distinct defender profiles coexist within a defensively effective professional squad.

### Anthropometric and motor-fitness profiles in context

The descriptive and correlation results highlighted strong interrelationships among anthropometric variables. High correlations between hip circumference, height, and weight, alongside strong associations between waist circumference, BMI, and body fat percentage, indicate that body size and composition cluster tightly in this cohort. The negative correlation between waist-to-hip ratio and hip circumference suggests that players with more favourable lower body profiles (larger hip girth relative to waist) tend to exhibit more advantageous body shape characteristics for stability and force transmission [10, 42, 43]. This clustering of anthropometric traits aligns with findings from recent studies showing that defenders with greater lower body girth and muscular development tend to perform better in physical contests and maintain postural stability during duels (e.g., in comparisons across professional leagues) [11, 16, 44]. The PCA findings extend these observations by demonstrating that body size and mass-related traits form a dominant latent dimension. The first principal component, dominated by height, weight, BMI, hip circumference and muscle mass, is consistent with a “body size/mass–composition” dimension commonly identified in profiling work and likely reflects the physical presence required for central defensive roles, especially in aerial duels and contact situations [21, 45, 46]. The second component, defined primarily by agility, Y-balance and coordination, appeared to represent an agility–balance dimension underpinning rapid changes of direction, recovery runs and maintenance of postural control in wide or covering roles [29, 47]. Such motor-fitness dimensions have been implicated in successful defensive coverage in dynamic match scenarios, with agility and balance underpinning recovery runs and lateral defensive adjustments in competitive contexts [30, 48]. The third component contrasted vertical jump power positively with body fat percentage and reaction time, reflecting an “explosiveness versus adiposity/slower response” dimension observed in other professional defender cohorts [49, 50], and is consistent with evidence that leaner physiques with optimised muscle mass are conducive to explosive lower-limb actions and faster perceptual–motor responses in match contexts [42, 51]. Together, these components reinforce the importance of evaluating defenders using integrated physical and neuromotor profiles rather than isolated metrics [52].

### Defender archetypes and ecological defensive efficiency

The three emergent defender archetypes provide a practically meaningful framework for understanding role specialisation within the defensive unit. High mass stoppers, characterised by greater BMI and muscle mass but slower agility, align with traditional central defenders whose primary tasks involve contesting aerial balls, winning duels, and defending set pieces [10, 17, 21, 53]. Mobile coverage defenders, who are leaner, quicker, and exhibit superior balance and vertical jump power, are more compatible with roles requiring recovery runs, channel coverage, and aggressive pressing in wide or advanced defensive zones [16, 25]. Balanced hybrids occupy an intermediate space, offering versatility rather than extreme specialization [21, 54]. Contemporary performance analyses in elite environments have similarly described such archetypal differentiation, where defenders are grouped functionally according to physical and movement-based profiles that map onto distinct tactical roles [10, 13, 26, 55]. However, given the modest sample size and single-club setting, these archetypes should be interpreted as descriptive patterns within this specific squad, rather than as definitive typologies generalisable to the wider league or other competitions. The significant group differences in muscle mass, body fat percentage and jump power were accompanied by medium-to-large effect sizes, whereas other contrasts showed negligible effects, reinforcing that the present study is best regarded as hypothesis-generating rather than confirmatory.

Although the ecological defensive indicators in this study were derived at the team level rather than the individual level, the context remains informative. Across the observed seasons, Enugu Rangers maintained a low rate of goals conceded per match while improving their defensive success index and points per goal conceded, indicating stable or improving team level defensive efficiency. Within this context, the coexistence of diverse defender archetypes reflects functional heterogeneity in physical and motor fitness profiles.

However, the ecological defensive indicators presented in this study are strictly team level descriptors and were not modeled as outcomes of individual player profiles. Because defensive efficiency was operationalised using aggregated team statistics rather than individual defensive events, the findings are subject to ecological inference limitations, and associations observed at the team level cannot be assumed to operate at the individual level. Therefore, the identified archetypes should be interpreted as structural descriptions of physical and motor fitness differentiation within a shared team environment rather than as explanatory determinants of defensive efficiency [56, 57].

This perspective aligns with ecological approaches to team performance, where heterogeneous role profiles within a defensive unit can enhance adaptability and collective robustness against varied offensive strategies [24, 39], although such interpretations remain conceptual rather than empirically tested in the current dataset. The findings support the use of archetype based profiling as a structured method for characterising defender role configurations within a team context and organising physical performance data in applied settings.

Any inference that specific archetypes contribute directly to team level defensive resilience would require integration of individual match event data, positional tracking metrics, and multilevel modelling approaches linking player characteristics to defensive actions and outcomes [29, 58], which is beyond the scope of the present ecological study.

### Practical implications for talent identification and conditioning

From an applied perspective, the profiling approach used in this study offers several practical benefits. First, the clear separation of archetypes suggests that defensive recruitment and talent identification can move beyond generic “good defender” labels toward targeted role profiles. Clubs could, for example, prioritise high-mass stoppers for central roles in systems that rely heavily on aerial dominance, while favouring mobile coverage defenders for high-pressing or transition-oriented tactics. In recruitment practice, using archetype profiles can help clubs align scouting metrics with specific tactical philosophies, reducing reliance on subjective assessments alone [7, 10, 19]. Second, the component and cluster structures point toward differentiated conditioning strategies. High-mass stoppers may benefit from programmes that preserve strength and power while improving agility and reaction time, reducing the performance cost of their larger body size. Mobile coverage defenders may require maintenance of speed, balance, and power while avoiding excessive increases in body mass that could compromise their core strengths. Balanced hybrids, meanwhile, can be developed strategically toward either profile depending on team needs. Such role-specific conditioning aligns with interdisciplinary conditioning frameworks advocating for tailored training prescriptions based on positional and functional demands [18, 19, 27]. Importantly, the integration of anthropometric, agility, balance, coordination, and jump-power metrics into routine testing could give coaching and performance staff a more nuanced view of each defender’s role suitability and developmental priorities.

### Methodological considerations and limitations

Several methodological issues and limitations must be emphasised to align interpretation with the strength and scope of the data. The study is based on a small sample of defenders from a single professional club, which restricts external validity. The principal components and clusters identified here may be sensitive to sampling variation and should not be assumed to represent stable structures across other teams or leagues. No formal cluster-stability procedures (e.g. bootstrapped clustering or split-sample validation) were undertaken due to the limited sample size; consequently, the archetypes should be considered exploratory, provisional and sample-specific. The design is ecological: anthropometric and motor-fitness data were collected at the individual level, whereas defensive performance indicators were aggregated at the team-season level. Player-specific match exposure (e.g. minutes played, starter vs. substitute status) and individual defensive actions (e.g. tackles, interceptions, blocks, pressures or expected-goals-against contributions) were not available. As a result, it is not possible to determine how much each defender, or each archetype, contributed to the observed defensive indicators, and all statements linking profiles to defensive performance must therefore remain associative and speculative. The defensive indicators themselves, while grounded in official statistics, are coarse grained: goals conceded per match, defensive success index, goal prevention rate, points per goal conceded and goal difference per match reflect combined effects of tactical organisation, goalkeeping, opponent quality and contextual match factors, and therefore provide a general representation of team defensive performance rather than isolating the specific impact of defenders or capturing nuances such as the quality of chances conceded, contextual match states or phase-of-play differences.Although statistical assumptions were checked and non-parametric tests were used where appropriate, the analysis remains exploratory and hypothesis-generating. The significant classification effects for muscle mass, body fat percentage and jump power showed medium-to-large η²ₕ values, whereas other contrasts had negligible effect sizes, indicating that these findings should be interpreted as signals warranting further investigation rather than definitive population estimates. Confidence in the multivariate structures should be interpreted in light of the modest sample and the absence of independent validation. Finally, the study was conducted in a data-constrained African professional context, which enhances the contextual relevance of the findings for similar environments where advanced tracking systems are not routinely available, but limits direct comparability with studies conducted in leagues where detailed positional and event data can be integrated with physical profiling.

### Directions for future research

Future research should extend this ecological profiling framework using richer datasets and broader samples. Multi-club and multi-league studies incorporating larger numbers of defenders would allow more robust evaluation of component and cluster stability and clarify whether similar archetypes emerge consistently across tactical systems and competitive levels. Integrating anthropometric and motor-fitness profiles with player-level match-tracking data such as GPS-derived movement metrics, event-level defensive actions and expected-goals-against models would enable multilevel analyses of how archetypes translate into on-pitch behaviour and measurable defensive events [29, 52, 58]. Methodologically, future work should incorporate formal cluster-stability and validation procedures (e.g. bootstrap and cross-validation approaches) and longitudinal designs that examine whether defenders transition between archetypes across seasons and how changes in physical and motor-fitness profiles relate to changes in individual and team-level defensive indicators. Such studies would move beyond the present hypothesis-generating analysis toward more definitive tests of how anthropometric and motor-fitness structures relate to defensive performance in professional football.

## Conclusion

This ecological profiling study characterises distinct anthropometric and motor fitness archetypes among professional defenders in an NPFL club and situates them within team-level defensive performance trends. The main contribution lies in applying multivariate profiling methods in a data-constrained African professional environment, and in explicitly integrating defender profiles with team-level ecological defensive indicators, rather than in proposing fundamentally new defender types. Findings are limited by the small single-club sample, the use of team-level defensive indicators rather than event-level actions, and the absence of formal cluster stability analysis, so archetypes should be interpreted as provisional and context-specific. These profiles may help practitioners in similar data-constrained environments to structure defender conditioning and role discussions, while recognising that causal links to defensive efficiency were not established.

## Supplementary Information

Supplementary Material 1.

## Data Availability

The datasets supporting the conclusions of this article are available in the University of Nigeria institutional repository and will be made easily available on request by the corresponding author, [cynthia.john@unn.edu.ng](mailto: cynthia.john@unn.edu.ng).

## Abbreviations

BMI: Body Mass Index
DSI: Defensive Success Index
GA: Goals Against
GA/PLD: Goals Conceded per Match
GD/PLD: Goal Difference per Match
GF: Goals For
GPR: Goal-Prevention Rate
ISAK: International Society for the Advancement of Kinanthropometry
KPI: Key Performance Indicator
NPFL: Nigeria Premier Football League
PCA: Principal Component Analysis
PPGC: Points per Goal Conceded
WHR: Waist–Hip Ratio
