# Supplementary material for: Anthropometric and motor-fitness signatures of defensive efficiency in professional football defenders: a principal component and cluster analysis
Source: BMC Sports Sci Med Rehabil. 2026 May 6;18:301. doi: 10.1186/s13102-026-01711-y (PMC13326548; doi:10.1186/s13102-026-01711-y)
Supplement: Supplementary file 1 — Supplementary Material 1. [file 13102_2026_1711_MOESM1_ESM.docx]

**Table 4: Normality Test (Shapiro-Wilk)**

| **Variable** | **W-Statistic** | **p-Value** |
| --- | --- | --- |
| Age | 0.8519 | 0.00025 |
| Height | 0.8812 | 0.00128 |
| Weight | 0.9194 | 0.01370 |
| BMI | 0.8773 | 0.00102 |
| Percentage body fat | 0.7077 | 4.92e-07 |
| Muscle mass | 0.7589 | 3.51e-06 |
| Coordination | 0.9101 | 0.00747 |
| Agility | 0.9315 | 0.03096 |
| Y balance | 0.8503 | 0.00023 |
| Vertical Jump Power | 0.9161 | 0.01105 |
| Reaction time | 0.9210 | 0.01527 |
